# Preoperative axillary lymph node staging by ultrasound-guided cytology using a four-level sonographic score

**DOI:** 10.1186/s12880-016-0116-1

**Published:** 2016-02-04

**Authors:** Caroline De Coninck, Jean-Christophe Noël, Rachel Boutemy, Philippe Simon

**Affiliations:** Erasme Hospital, 808 route de Lennik, 1070 Anderlecht, Belgium

**Keywords:** Axillary lymph nodes, Breast cancer, Ultrasound, Cytology, Metastasis

## Abstract

**Background:**

The staging of axillary lymph nodes is critical to the management and prognosis of breast cancer, the most frequent cancer in females. Neoadjuvant therapy and lymph node dissection are recommended when malignant cells invade the lymph nodes. Therefore the pre-operative examination of these lymph nodes is crucial to treatment.

**Methods:**

In this study, we examined the effectiveness of cytology through ultrasound-guided fine needle aspiration (USG-FNA) and ultrasound (US) imaging using an established classification system in correctly identifying lymph node status compared to the final histological results after surgery.

**Results:**

Cytology by USG-FNA and US classification were found to be promising methods of axillary lymph node staging.

**Conclusions:**

US and CB offer minimally invasive techniques to pre-operatively examine these lymph nodes in patients with primary breast cancer.

## Background

Breast cancer is the most frequent female cancer [1] and the second leading cause of female cancer death in Belgium [2]. Therefore, breast cancer management is critically important. When an axillary lymph node is invaded, neoadjuvant chemotherapy and axillary lymph node dissection are indicated [3, 4]. In terms of survival, it is now widely accepted that there are advantages of neoadjuvant chemotherapy in patients with node-positive breast cancer [3, 4]. Axillary lymph node status is an important factor in the prognosis and management of breast cancer. Several methods to detect positive axillary lymph nodes during the pre-operative diagnosis have been evaluated, including ultrasound-guided fine needle aspiration (USG-FNA) cytology, ultrasound-guided biopsy and, as an imaging method, axillary ultrasound [5]. If a positive lymph node is not found during the pre-operative evaluation, a less invasive sentinel node biopsy will be utilized instead of axillary lymph node dissection [6]. The goal of this study was to compare the results of axillary lymph node status by cell-block obtained through fine-needle aspiration [7] and by axillary ultrasound, according to a classification system derived from Stavros [8].

## Methods

### Patient dataset

The Hôpital Erasme ethics committee approved this study. All the results were analyzed retrospectively and anonymously.

This study included a series of 208 cell or cytoblocks (CB) of axillary or para-axillary (chest wall, subclavicular and intra-mammary) lymph nodes from 184 patients (141 patients with one, 18 patients with two, one patient with three, and one patient with four evaluated lymph nodes) with primary breast cancer from a university center collected between October 2008 and August 2012.

Fine-needle aspirations were performed on all patients by a radiologist using a 21-gauge, ultrasonographic (US)-guided needle prior to any operative procedure on the breast or axilla, regardless of whether the nodes appeared normal or abnormal.

### Lymph node classification

Lymph nodes were classified into four categories according to criteria derived from Stavros’ classification: normal “N”, minimally suspect “+”, mildly suspect “++” and highly suspect “+++”. The details of these classifications are as follows:

- “+”: lymph node with a maximum size of cortical thickness of 3 mm with regular capsular thickening
- “++”: lymph node with irregular capsular thickening (with notches) or with regular capsular thickening and cortical thickness greater than 3 mm in size
- “+++”: complete loss of lymph node structure; irregular cortex or absence of lymph node hilum.

**Fig. 1:**
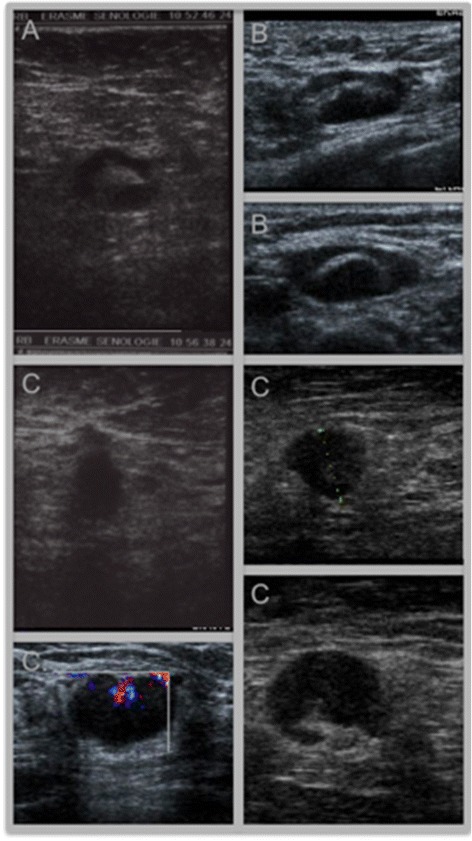
Sonographic images of a minimally suspect axillary lymph node “+” (**A**), a mildly suspect axillary lymph node “++” (**B**) and highly suspect axillary lymph node “+++” (**C**)

Normal or “+” lymph nodes were considered non-suspicious, and “++” and “+++” lymph nodes were considered suspicious (Fig. 1).

### Cytoblock and cytospin techniques

The sample obtained by US-guided fine-needle aspiration (USG-FNA) was placed in Saccomanno fluid and sent to the laboratory where it was separated into two samples: one for the CB technique and one for the cytospin technique. Both samples were centrifuged at 1400 rpm for 10 min. The cytospin sample was placed in a Shandon EZ Cytofunnel® with a few drops of concentration solution, then placed in the Shandon Cytospin®4 centrifuge for 10 min at 500 rpm. It was then placed on a microscope slide for cytologic analysis. For CB, 2–4 drops of Reagent 2 from the cytoblock kit were added to the pellet, and it was resuspended and incubated for one minute. Two to four drops of Reagent 1 from the kit were added, causing polymerization. After one minute, the resulting polymerized material was placed between two sponges in a standard cassette and routinely processed with other biopsies and paraffin-embedded blocks. Four-micron sections were cut and stained with hematoxylin and eosin. Immunohistochemistry (IHC) was performed on each cytoblock using broad-spectrum cytokeratin primary antibodies (clone AE1-AE3, dilution: 1/100, Dakocytomation, Glostrup, Denmark).

The CB results were classified into one of three categories: “C1” inadequate, “C2” negative and “C5” malignant.

### Histological assessment

**Fig. 2:**
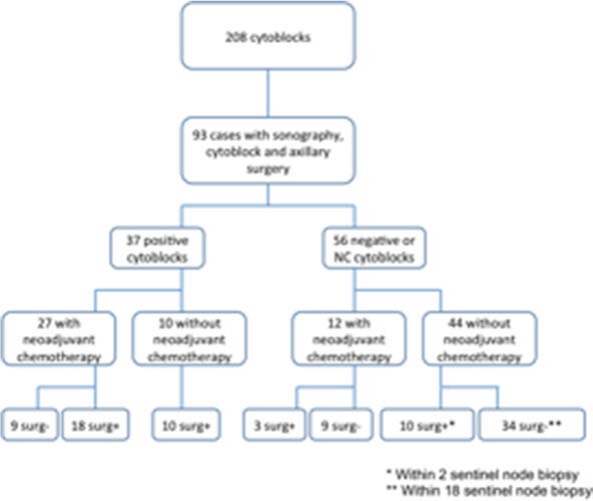
Two hundred and eight cytoblocks (CB) were performed at Erasme Hospital between October 2008 and August 2012. Of these, 93 cases had sonography, cytoblock and axillary surgery. Of the CBs performed, 37 were positive and 56 were negative or non-contributory (NC). Of the 37 positive CB, 10 did not receive neoadjuvant therapy. Of the 56 negative or NC cytoblocks, 44 did not receive neoadjuvant chemotherapy

From the 208 CBs, only 93 had final histological confirmation of axillary lymph nodes after axillary lymph node dissection or sentinel node biopsy. These were categorized as either negative, when all of the examined lymph nodes were negative for metastasis, or positive, when there was evidence of metastasis in one or more lymph nodes. The same histopathologist performed histological assessments. Of the 93 results, only 54 had no neoadjuvant chemotherapy and were thus interpretable (Fig. 2).

The results were analyzed according to the type of breast cancer, the size of the evaluated lymph nodes, type of surgery (sentinel node biopsy or axillary lymph node dissection), surgeon, radiologist, patient’s age, patient’s BMI, and the presence or absence of neoadjuvant chemotherapy.

## Results

Ninety-three CBs from 80 patients who underwent surgery had histological confirmation of axillary lymph nodes after sentinel node biopsy or axillary lymph node dissection. The 80 patients were between 28 and 84 years old. Tumor size was between 4 and 85 mm, and the axillary lymph node size was between 5 and 42 mm. In 73 cases, an axillary lymph node dissection was performed, and in 20 cases a sentinel node biopsy was performed.

Because 39 patients received neoadjuvant chemotherapy, the histological results from their surgeries were not interpretable. In effect, only the 54 patients who did not receive neoadjuvant chemotherapy were analyzed. Among these, 15 had non-suspicious lymph nodes (N), 23 were minimally suspect, nine were mildly suspect, and seven were highly suspect (Table 1). Five CBs were inadequate, 39 were negative, and 10 were positive (Table 2).

**Table 1:** Lymph nodes histological results according to pre-operative sonographic results

	Histological results from surgery			
Echography		Negative (%)	Positive (%)	Total
	Echo N	14 (93)	1 (7)	15 (100)
	Echo +	18 (78)	5 (22)	23 (100)
	Echo ++	2 (22)	7 (78)	9 (100)
	Echo +++	0	7 (100)	7 (100)
	Total	34 (63)	20 (27)	54 (100)

**Table 2:** Lymph nodes histological results according to pre-operative cytoblock results

	Histological results from surgery			
Cytoblock		Negative (%)	Positive (%)	Total
	C 1	2 (40)	3 (60)	5 (100)
	C 2	32 (82)	7 (18)	39 (100)
	C 5	0	10 (100)	10 (100)
	Total	34 (63)	20 (37)	54 (100)

Of the 54 cases without neoadjuvant chemotherapy, 34 had a negative histological result and 20 had a positive histological result. Of the 34 negative results, 14 had an echo of N, 18 echo of + (also considered non-suspicious) and only two had an echo of ++. Of the 20 positive results, one had an echo N, five had + echoes, seven had ++ echoes, and seven had +++ echoes.

Of the 54 cases without neoadjuvant chemotherapy, 34 had a negative histological result and 20 had a positive histological result. Of the 34 negative results, only 2 had non-contributory CB (C1), 32 had negative CB (C2) and there were no positive CB (C5). Of the 20 positive results, 3 had non-contributory CB (C1), 7 had negative CB (C2) and 10 had positive CB (C5).

The final histological result was negative for 34 cases and positive for 20 cases.

Of the 38 non-suspicious lymph nodes based on US (N or “+”) in patients who did not have neoadjuvant therapy, lymph node metastasis was found in six cases (15.8 %), whereas there were no malignant cells in 32 cases (84.2 %) (Table 1).

Of the 16 suspicious lymph nodes based on US (“++” or “+++”) in patients who did not receive neoadjuvant therapy, lymph node metastasis was found in 14 cases (87.5 %), and no malignant cells were detected in two cases (12.5 %) (Table 1).

All metastatic axillary lymph nodes identified by CB preparation in the absence of neoadjuvant chemotherapy (C5) had positive final histological results (100 %) (Table 2).

Of the 39 axillary lymph nodes in which metastasis was not found by CB preparation (C2), 32 had negative final histological results in the absence of neoadjuvant chemotherapy (82 %), and seven had positive final histological results (18 %) (Table 2).

The results of the pre-operative lymph node staging by US and by CB preparation with the final histological result were compared using a *χ*^2^ statistical test. A statistically significant correlation was found between the results in the two cases (Table 2). The results obtained were statistically significant (*χ*^2^ 23.691 (*p* = 0.00000717) for CB and *χ*^2^ 25.381 (*p* = 0.00000046) for US.

Of the C5 lymph nodes (10 cases) or those found by US to be suspicious (9 “++” and 7 “+++”) in the pre-operative evaluations (26 cases), only two cases of “++” were ultimately negative in the absence of neoadjuvant therapy in the final histological results.

To determine whether some of the variables could explain a discordance in the results, or whether the rate of contribution changed depending on these variables, the impact of the variables on the results were analyzed using a *χ*^2^ statistical test. None of the variables were found to be statistically significant.

In comparing the results of the CB preparation to those of the cytospins (57 cases), only three cases were discordant: three cases of inadequate CB had cytospin results of C3, C4 or C5.

A comparison of axillary lymph node status between CB preparation or US, and the final histological results of lymph nodes after surgery in the absence of neoadjuvant chemotherapy, indicated that false-negative results for metastasis were identified by CB in seven cases (7/39, 18 %) compared to one “N” (1/15, 6.67 %) and five “+++” (5/23, 21.7 %) cases obtained by US alone (6/38, 15.8 %). Moreover, the CB technique showed 0 % false-positive cases compared to 12.5 % (two “++”, 16 “++”, and “+++”) false-positive cases obtained by US alone. The results summarized in Table 3 show the sensitivities, specificities, positive predictive values (PPV) and negative predictive values (NPV) for US alone compared to CB, excluding CB cases with inadequate results, and patients who received neoadjuvant chemotherapy.

**Table 3:** Statistical peformance

	Sensibility (%)	Specificity (%)	PPV (%)	NPV (%)
Echography*	68.4	94.1	86.7	84
Cytoblock	58.8	100	100	82.1
Echography* + Cytoblock	75.0	94.1	88.2	86.5

^*^ Echography N and + considered as non suspicious; echography ++ and +++ considered as suspicious

Based on the values displayed in Tables 1 and 2, we calculated the sensitivity, specificity, positive predictive value (PPV) and negative predictive value (NPV) of echography* and cytoblock both alone and together. *Echoes of N and + were considered non-suspicious; echoes of ++ and +++ considered suspicious.

For the “++” and “+++” lymph nodes classified as suspicious in the absence of neoadjuvant therapy, the sensitivity of CB in the 54 cases was 58.8 % compared to 68.4 % for US. The specificity (100 % versus 94.1 %), PPV (100 % versus 86.7 %) and NPV (82.1 % versus 84 %) values were high for both diagnostic methods (Table 3).

In cases where only the “+++” lymph nodes were considered suspicious, the sensitivity of CB in the 54 cases was 58.8 % compared to 85.7 % for US. Specificity was 100 % versus 100 %, PPV was 100 % versus 100 %, and NPV was 82.1 % versus 93.3 % (Table 3).

For non-suspicious lymph nodes with N or “+” results from US and C2 (a combination of the CB and US techniques) without neoadjuvant therapy, the sensitivity was 75 %, specificity was 94.1 %, PPV was 88.2 % and NPV was 86 % (Table 3).

These results support the hypothesis that pre-operative CB from USG-FNA biopsy of axillary lymph nodes, and axillary US using a modified version of Stavros’ classification, are two promising methods of pre-operative axillary staging, with axillary US being the preferred means of staging.

## Discussion

This study is based on a series of CBs from USG-FNA and sonography of axillary lymph nodes in patients with primary invasive breast cancer. Among non-invasive approaches, US and USG-FNA cytology have been reported to have high accuracy for staging axillary lymph nodes. Published estimates of USG-FNA cytology show a sensitivity varying from 25 to 87 %, a specificity ranging from 14 to 100 %, an NPV between 54 and 78 % and a PPV ranging from 37 to 100 % [6, 7, 9–13]. The results obtained in our study are similar to and even exceed these published findings. The only study to have previously described results from the CB technique, Engohan et al., has results very similar to those described here, with a better sensitivity (73 % versus 58 %), a lower NPV (78 % versus 82 %) and same specificity and PPV (100 %). Analysis of our results showed that for patients with positive nodes by CB (C5) or positive nodes by US (“+++”), the probability of having axillary nodal metastasis upon surgery in the absence of neoadjuvant therapy is 100 % (PPV). Similarly, for patients with negative nodes by the CB technique and by US (N or “+”), the probability of having no axillary nodal metastasis upon surgery is 86.5 % (NPV) in the absence of neoadjuvant chemotherapy.

We found seven false-negative cases by the CB technique and by US, including one that had an inadequate CB result. Another false-negative lymph node was evaluated simultaneously with an ipsilateral lymph node that was positive at pre-operative staging; surgery showed two positive lymph nodes out of 12 analyzed. In effect, the second node was confirmed as positive without insurance, because the pathologist could not prove which one was the one diagnosed before surgery. We also found two false-positive cases by US when the CB result was negative, and the size of the pre-operative lymph node, as determined by US, was not the same as that measured by the pathologist after surgery.

This analysis reveals some limitations to our study. First, it is possible that the lymph node analyzed by the pathologist after surgery (sentinel node biopsy or axillary lymph node dissection) was not the same one as that evaluated by US and USG-FNA during pre-operative staging.

Additionally, there were 39 cases of lymph nodes from patients who had received neodajuvant chemotherapy. For those cases, it is not possible to compare the pre-operative and final histological results.

## Conclusion

Axillary lymph node status is an important component of staging and treatment planning in breast cancer. Our study confirms that the use of a combination of US and CB may improve the evaluation of axillary lymph nodes in patients with primary breast cancer. These techniques are simple, inexpensive, minimally invasive, and allow for immunostaining. They also permit the referral of patients with primary breast cancer to neoadjuvant chemotherapy if the CB result is C5 or if the US result is “+++”. In the same way, for patients with a CB result of C2 and US result N or “+”, these techniques may preclude the use of neoadjuvant chemotherapy. Given the limited number of cases in our study, additional, new studies will be necessary.

## Abbreviations

CB: cytoblock
IHC: immunohistochemistry
NPV: negative predictive value
PPV: positive predictive value
US: ultrasound
USG-FNA: ultrasound-guided fine needle aspiration
